# Voice changes associated with stress and distress in people living with diabetes: Results from the Colive Voice study

**DOI:** 10.1371/journal.pdig.0001689

**Published:** 2026-08-31

**Authors:** Charline Bour, Abir Elbéji, Dulce Canha, Noémie Topalian, Hanin Ayadi, Christelle Godin, Claire Guyon-Gardeux, Pierre-Yves Benhamou, Malvina Billères, Guy Fagherazzi

**Affiliations:** 1 Université Grenoble Alpes, Fonds de Dotation Clinatec, Grenoble, France; 2 Deep Digital Phenotyping Research Unit, Department of Precision Health, Luxembourg Institute of Health, Strassen, Luxembourg; 3 University of Luxembourg, Esch-sur-Alzette, Luxembourg; 4 Urban Development and Mobility, Luxembourg Institute of Socio-Economic Research, Porte des Sciences, Maison des Sciences Humaines, Esch sur Alzette/Belval, Luxembourg; 5 Université Grenoble Alpes, CEA-Leti, Grenoble, France; 6 Université Grenoble Alpes, Inserm, CHU Grenoble Alpes, Grenoble, France; Massachusetts Institute of Technology, UNITED STATES OF AMERICA

## Abstract

Stress and diabetes distress impact glycemic control, self-care, and health outcomes in people living with diabetes, but current diabetes technologies lack easy-to-use, non-invasive methods to detect these states. The objective was to identify vocal features associated with stress and diabetes distress in people living with diabetes. Thus, we analyzed data from 679 adults with diabetes (381 women, 298 men), recruited via the global vocal biomarker screening platform *Colive Voice*. Participants recorded a standardized 30-second text. Vocal features were extracted using DisVoice across phonation, prosody, articulation, and phonological domains. Associations between vocal features and stress (self-reported, scale: 0–4) and diabetes distress (Problem Areas in Diabetes (PAID) questionnaire; categories: PAID<20, 20 ≤ PAID<40, 40 ≤ PAID<60, PAID≥60) were analyzed separately for men and women. Multivariate ordinal logistic regression models were adjusted for age, language, diabetes type, and HbA1c, with False Discovery Rate correction.In women, results suggest that stress was associated with 25 voice features across all domains, while distress was linked to one articulatory feature, suggesting broader effects of stress. In men, both stress (25 features) and distress (18 features) affected multiple domains, but with distinct acoustic patterns. No voice features were shared between stress and distress. These patterns were consistent across age, diabetes type, and language.To conclude, voice changes reflect stress and diabetes distress in people with diabetes, each characterized by distinct acoustic features and additional sex-specific signatures. These findings highlight the potential of vocal biomarkers to differentiate and monitor these emotional states, supporting more timely and personalized interventions to improve diabetes outcomes.

## Author summary

Stress and diabetes distress are common in people living with diabetes and can worsen blood sugar control and overall health. Currently, detecting these emotional states relies on questionnaires that are completed only at specific clinical visits, making it difficult to capture how they impact diabetes management. In this study, we explored whether voice recordings collected via smartphone could reflect stress and diabetes distress in adults with diabetes. We analyzed speech recordings from 679 participants and identified acoustic features of the voice that were statistically associated with each emotional state. We found that stress affected a broader range of vocal characteristics than diabetes distress, and that the two outcomes produced distinct vocal patterns with no overlapping features. We also found that these patterns differed between men and women, suggesting that vocal signatures of stress and distress in diabetes are sex-specific.

## Introduction

Stress is a natural psychological and physiological reaction to challenges and threats [1]. It can be acute (such as a short-lived response to an argument), or chronic, resulting from prolonged or repeated exposure to stressors (such as unemployment). Stress activates the body’s stress response system, triggering the release of several hormones, including cortisol and adrenaline, which can significantly affect overall health [1–3]. Together, these hormones prepare the body to respond quickly and effectively to immediate threats [4]. While this response is adaptive in the short term, repetitive or chronic activation can have consequences on health, such as a higher risk of cardiovascular disease, depression, and impaired immune function [5–9]. Several studies also highlighted a direct link between chronic stress and the development of type 2 diabetes, because of persistent hyperglycemia and insulin resistance [10–13]. For people living with diabetes, this hormonal response can be particularly challenging. Stress-induced increases in blood glucose levels can interfere with glycemic control. They may lead to acute complications, such as hyperglycemic episodes, hypoglycemic episodes, and potentially to long-term consequences like cardiovascular disease or neuropathy [14–16].

In addition to general life stress, people living with diabetes can experience diabetes distress, an emotional burden related to the ongoing demands of living with diabetes [17]. Diabetes distress encompasses several domains, including emotional distress, fear of complications, social distress, eating distress, management distress, and diabetes burnout. Diabetes distress can affect any patient at some point, with higher rates in type 1 diabetes and among those with poor glycemic control, recent diagnosis, or complications [18–21]. High levels of distress are associated with lower motivation for self-care, reduced treatment adherence, and poorer glycemic control, contributing to worsened health outcomes, which in turn aggravate emotional strain [22–25].

Thus, while distinct issues, stress and diabetes distress share a common and significant impact on the health of people with diabetes. Both can lead to elevated blood glucose levels, lower engagement in self-care behaviors, and poorer health outcomes. They also may tend to reinforce each other, creating a vicious circle where emotional strain worsens metabolic control, which in turn intensifies psychological distress.

In clinical research, these emotional states are rarely evaluated. When they are, they are assessed using self-reported questionnaires, such as the Perceived Stress Scale (PSS) for stress [26] and the Problem Areas in Diabetes (PAID) scale for diabetes distress [27]. While these tools are valuable for screening, they rely on retrospective recall and subjective interpretation, which can introduce bias and fail to capture the real-time, dynamic fluctuations of an emotional state. Moreover, questionnaires are usually administered at discrete time points and might miss short-term fluctuactions in emotional states. Given the dynamic and unexpected nature of stress and distress and their consequences on the health of people with diabetes, there is a need for tools that can continuously and non-invasively assess these emotional burdens in real-time. Voice recordings can be collected frequently and with minimal effort using already available devices (such as smartphones), offering the possibility of more continuous and ecologically valid assessments.

We hypothesize that voice could be used as a non-invasive and complementary indicator of stress and distress in people with diabetes. Rather than replacing validated questionnaires, voice analysis may offer an additional low-burden source of information that captures short-term fluctuations in emotional states. Changes in voice features (such as tone, pitch, speech rate, and prosody) have been shown to correlate with stress and emotions [28–32]. Voice analysis could therefore provide a real-time window into emotional strain, enabling individuals with diabetes and healthcare professionals to detect and respond to stress or distress as it occurs, thereby preventing complications.

## Methods

### Ethics statement

Colive Voice is registered on ClinicalTrials.gov (NCT04848623) and was approved by the National Research Ethics Committee of Luxembourg (study number 202103/01) in March 2021. All participants provided informed consent to take part in the study.

### Data availability

The data underlying the findings of this study are publicly available at: https://github.com/LIHVOICE/VoiceStressDistress. The dataset consists of anonymized voice features and associated metadata derived from the Colive Voice project (Luxembourg Institute of Health, Luxembourg; ClinicalTrials.gov Identifier: NCT04848623). The authors obtained permission to use the Colive Voice data for the present analysis through a collaboration agreement with the Luxembourg Institute of Health.

### Study population

Colive Voice is a global and multilingual screening platform for vocal biomarkers, designed to support the detection and monitoring of various chronic diseases and common health symptoms [33]. The study is open to individuals aged 15 years and older and collects data in several languages. Each participant provides informed consent and contributes by completing several questionnaires and performing standardized vocal tasks using their personal devices (smartphones or computers). In this work, we included participants who (i) reported having diabetes, (ii) completed PAID questionnaire and answered to the stress question (“Stress means a situation in which a person feels tense, restless, nervous or anxious or is unable to sleep at night because his/her mind is troubled all the time. Do you feel this kind of stress these days?”), and (iii) provided a usable voice recording for the standardized reading task. All recordings underwent a manual quality control procedure prior to analysis. Each audio file was reviewed and categorized based on its quality (e.g., clean, moderate noise, extreme noise). Recordings with insufficient quality (e.g., extreme noise, absence of usable speech, corrupted audio) were excluded from further analysis. Recordings with moderate noise or minor issues (e.g., excess silence or background noise) were retained when speech remained clearly intelligible, and were preprocessed accordingly (e.g., trimming of silence or noise segments). In all, 843 recordings underwent quality control and 679 (81%) were included for analysis.

This procedure ensured a balance between data quality and ecological validity and that extracted acoustic features were reliable and comparable across participants.

For this analysis, we included 679 participants with type 1 and type 2 diabetes and analyzed data separately by sex. Each participant contributed a single voice recording corresponding to a standardized task. Recordings were performed in either French or English, depending on the participant’s preferred language. The task used in this work is reading the 25th article from the Universal Declaration of Human Rights. Participants also provided self-reported information such as age and recent HbA1c levels. They were asked to rate how stressed they had been feeling in recent days, which was used to compute a stress score ranging from 0 (not at all) to 4 (very much). All participants who reported having diabetes were automatically given the PAID questionnaire, allowing the calculation of the diabetes distress score. The validated threshold to define high levels of distress is a PAID score equal to or above 40. We decided to group PAID scores into four categories (Low distress: 0 ≤ PAID<20, Moderate distress: 20 ≤ PAID<40, High distress: 40 ≤ PAID<60, and Very high distress: PAID≥60) to facilitate analysis and comparison between groups. This simplifies the interpretation of distress levels, facilitating inter-group comparison and understanding associated risk factors (HbA1c, type of diabetes, age). No imputation was performed, as only participants with complete questionnaire data and usable recordings were included in the analyses.

### Voice feature extraction

We used the open-source software DisVoice version 0.1.8 on Python 3.8.20 to extract features from the recordings. Feature extraction was performed using the default settings provided by the library, including prosodic [34,35], phonatory [35,36], phonological [37] and articulatory [35,38] feature sets [34,35]. Prosodic features capture aspects such as intonation, rhythm, and speech rate. Articulatory features such as formant frequencies and their dynamics reflect how speech sounds are physically produced. Phonatory features relate to the functioning of the vocal folds and include measures such as jitter, shimmer, and harmonics-to-noise ratio, markers of voice quality. Phonological features capture the broader organization and use of sound units in speech, providing insight into linguistic processing or articulation patterns.

We used the mean and standard deviation of each feature across the vocal task to summarize both central tendency and variability in speech production. This approach allows for a compact representation of each recording and reduces dimensionality, which is important given the large number of extracted features relative to the sample size. It also facilitates the use of statistical models while limiting the risk of overfitting. While some features, such as MFCCs, capture time-varying properties of speech, aggregating them provides a global characterization of vocal behavior that has been commonly used in similar studies. However, this approach does not preserve temporal dynamics within recordings. This resulted in the extraction of 45 prosodic features, 16 phonation features, 246 articulation features, and 38 phonological features.

### Data analysis

The analysis aimed to identify voice features significantly associated with stress and distress levels among female and male participants with diabetes. Analyses were stratified by sex due to physiological differences in vocal production. In particular, differences in vocal fold anatomy, pitch range, and hormonal influences are known to affect acoustic properties of speech and may lead to sex-specific patterns in how emotional states are expressed through voice [39, 40]. To ensure that associations were not driven by random sampling fluctuations, we applied a repeated subsampling procedure. For each feature category (prosody, phonation, articulation, and phonological), datasets were randomly subsampled ten times (90% of the data retained at each iteration). Within each subsample, features were standardized, and univariate ordinal logistic regression models were fitted to evaluate associations with the outcomes, adjusting for age, language, type of diabetes, and HbA1c (1 if HbA1c ≥ 7, 0 otherwise) to account for potential differences in vocal characteristics across linguistic groups. 345 acoustic features were tested across four feature domains. For each outcome (stress and diabetes distress) and each sex group, analyses were repeated across 10 subsampling iterations, resulting in a large number of statistical tests. P-values were adjusted within each analysis using False Discovery Rate correction to account for multiple comparisons. Features were retained if they were significant in at least 7 out of 10 subsampling iterations, consistent with the stability selection framework of Meinshausen and Bühlmann, who recommend selection thresholds in the range of 0.6 to 0.9 [41]. Alternative thresholds were examined: 6/10 proved overly permissive, retaining features with weak associations, while thresholds of 8/10 and 9/10 were excessively restrictive given that multiple testing was already controlled via FDR correction at each iteration, risking unnecessary loss of genuine signal. A threshold of 7/10 was therefore selected as the most appropriate balance between reproducibility and sensitivity. Final ordinal logistic regression analyses were then conducted on the whole males and females datasets to compute beta coefficients and odds ratios, representing the strength and direction of each association, to investigate the magnitude of impact of stress and distress on voice features.

## Results

### Population characteristics

The study population consisted of 679 participants, 298 males and 381 females, with a similar mean age across sex (44.2 ± 16.8 years for males, 45.2 ± 15.3 years for females). Table 1 describes the overall characteristics of the study population. Approximately one-third of participants had type 1 diabetes (36.2% of males, 34.9% of females), and around two-thirds were English speakers. Stress levels varied across the population, with the majority (78,3% in males, 73,8% in females) reporting low to moderate stress. The average HbA1c levels were comparable between sexes (7,6% ± 2,4% for males, 7,4% ± 2,2% for females). The mean PAID score was slightly higher among females (35,5 ± 25.6) than males (32.3 ± 25.4). On average, participants with type 1 diabetes had a higher PAID score (males: 46.8 ± 23,9, females: 46.3 ± 25.5) compared to those with type 2 diabetes (males: 24 ± 22.3, females: 29.7 ± 23.8).

**Table 1:** Study population characteristics.

	Males (N = 298)	Females (N = 381)
**Demographics**		
**Age in years (mean ± std)**	44,2 (±16,8)	45,3 (±15,3)
**Ethnicity (n, %)**		
White/Caucasian	175 (58,7%)	265 (69,6%)
Black/African/Caribbean	77 (25,8%)	78 (20,5%)
Hispanic/Latino	12 (4%)	9 (2,4%)
Asian	15 (5%)	8 (2,1%)
Mixed/Multiple Ethnic Group	8 (2,7%)	8 (2,1%)
Arab	2 (0,7%)	1 (0,3%)
Don’t know	1 (0,3%)	2 (0,5%)
Other	8 (2,7%)	10 (2,6%)
**Education (n, %)**		
Bachelor’s Degree	120 (40,3%)	143 (37,5%)
High School	104 (34,9%)	118 (31%)
Master’s Degree	28 (9,4%)	66 (17,3%)
Some High School or lower	24 (8,1%)	31 (8,1%)
PhD or higher	19 (6,4%)	17 (4,5%)
Prefer not to say	3 (1%)	6 (1,6%)
**Diabetes characteristics**		
**Type 1 diabetes (n, %)**	108 (36,2%)	133 (34,9%)
**Diabetes duration T1D / T2D in years (mean ± std)**	T1D: 11,6 (± 10,1) T2D: 10,2 (± 8,4)	T1D: 14,8 (± 11,9) T2D: 8,55 (± 7,46)
**HbA1c in % (mean ± std)**	7,6% (± 2,4%)	7,4% (± 2,2%)
**Diabetes treatment (n, %)**		
Use insulin	154 (51,7%)	174 (45,7%)
Use oral medication	162 (54,4%)	193 (50,7%)
Use CGM	95 (31,9%)	124 (32,5%)
**Language**		
**English speakers (n, %)**	201 (67,4%)	294 (62,5%)
**Outcomes**		
**Stress (n, %)**		
0 - Not at all	86 (28,9%)	69 (18,1%)
1 - A little	78 (26,2%)	115 (30,2%)
2 - To some extent	69 (23,2%)	97 (25,5%)
3 - Rather much	44 (14,8%)	64 (16,8, %)
4 - Very Much	21 (7%)	36 (9,4%)
**PAID**		
Total (mean ± std)	32,3 (± 25,4)	35,5 (± 25,6)
0 ≤ PAID<20 - n (%)	122 (40,9%)	134 (35,2%)
20 ≤ PAID<40 - n (%)	59 (21,8%)	93 (24,4%)
40 ≤ PAID<60 - n (%)	65 (19,8%)	73 (19,2%)
PAID≥60 - n (%)	52 (17,4%)	81 (21,3%)

The study population included participants with diverse ethnic and educational backgrounds, as well as varying diabetes characteristics and treatment profiles (Table 1). Moreover, in women, stress levels were slightly higher in individuals with type 1 diabetes (Mean = 1.85, SD = 1.17) compared to type 2 diabetes (M = 1.41, SD = 1.26). Diabetes distress (PAID scores) was higher in type 1 diabetes (M = 46.33, SD = 25.47) than in type 2 diabetes (M = 29.67, SD = 23.82). A similar pattern was observed in men, with higher stress in type 1 diabetes (M = 1.68, SD = 1.22) compared to type 2 diabetes (M = 1.05, SD = 1.18), and higher PAID scores in type 1 diabetes (M = 46.84, SD = 23.92) than in type 2 diabetes (M = 24.03, SD = 22.33).

**Fig 1:**
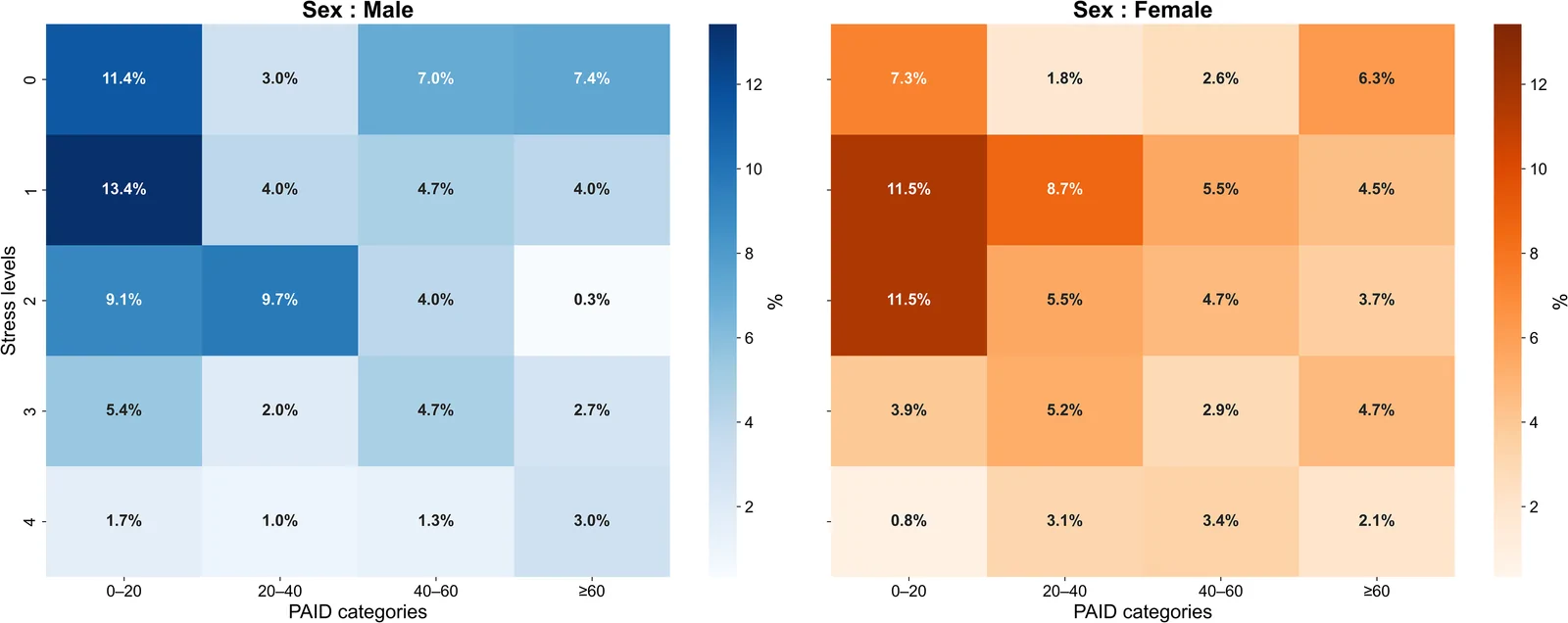
Cross-distribution of perceived stress levels and diabetes distress (PAID categories) by sex. Each cell shows the percentage of participants falling into a specific combination of stress level (0-4) and PAID category (0-20, 20-40, 40-60, ≥ 60).

The cross-distributions of stress levels and PAID score categories were computed separately for males and females (Fig 1). These tables reveal a high heterogeneity of psychological profiles within the population. Some participants reported high stress with low distress, or high distress with low stress, while others experienced both simultaneously. These observations suggest that stress and diabetes-related distress are not redundant constructs, but rather reflect distinct aspects of the psychological experience of people living with diabetes. This is confirmed by Spearman’s correlations, which showed weak and non-significant associations both in women (0.08, p = 0.11) and in men (0.01, p = 0.85). These findings support our decision to analyze both dimensions independently in order to identify vocal features specifically associated with each.

### Voice features associated with stress in people with diabetes

The following associations correspond to acoustic features retained through the stability-based selection procedure described in the Methods section. Specifically, features were considered robust if they were significant in at least 7 out of 10 subsampling iterations after False Discovery Rate correction, and were subsequently confirmed in the final models estimated on the full dataset.

#### Females.

For female participants, stress levels were significantly associated with a set of 25 acoustic features across prosodic, phonatory, articulatory, and phonological categories (see Table 2). Among prosodic features, higher stress was linked to reduced pitch and energy variability (e.g., F0msestd, OR = 0.70; stdlastEvoiced, OR = 0.67), shorter and more uniform voiced segments (avgdurvoiced, OR = 0.73; stddurvoiced, OR = 0.64), and altered voicing patterns (VVU, OR = 0.65; UVU, OR = 1.67). These suggest that stressed speech is less dynamic and more constrained in temporal and energetic structure. Phonation-related changes included increased shimmer (avg Shimmer, OR = 1.53), indicating unstable vocal fold vibration under stress. Articulatory features such as avg DF2 (OR = 0.62) and avg DDMFCCon_7 (OR = 0.62) decreased with stress, indicating less pronounced formant transitions and spectral dynamics, which may reflect reduced articulatory precision. In contrast, variability-based features like std DDMFCCon_8 (OR = 1.74) increased, possibly reflecting irregular articulatory adjustments. Regarding phonological features, nasal (nasal_mean, OR = 0.65) and close vowel sounds (close_mean, OR = 0.75; close_std, OR = 0.67) decreased with stress, suggesting altered vowel articulation. Meanwhile, increased energy in stop consonants (stop_mean, OR = 1.54) and reduced anterior vowel clarity (anterior_mean, OR = 0.78) suggested potential speech simplification aimed at reducing articulatory effort.

**Table 2:** Voice features associated with stress in women with diabetes.

Category	Feature	Feature meaning	Coefficient (SD)	Odds Ratio [CI 95%]	PValue (FDR corrected)
Prosody	avgdurvoiced	Average duration of individual voiced segments, reflects phonation stability and continuity	-0.308 (0.098)	0.73 [0.61 - 0.89]	0.002
	VVU	Proportion of voiced speech over total	-0.436 (0.1)	0.65 [0.53 - 0.79]	p < 0.001
	stdlastEvoiced	Standard deviation of energy in final voiced segments; captures variability in speech endings	-0.394 (0.109)	0.67 [0.54 - 0.84]	p < 0.001
	avgtiltEvoiced	Average slope of energy change in voiced segments	-0.448 (0.094)	0.64 [0.53 - 0.77]	p < 0.001
	stddurvoiced	Variation in the duration of voiced segments	-0.44 (0.102)	0.64 [0.53 - 0.79]	p < 0.001
	avgEunvoiced	Average energy in unvoiced segments	-0.405 (0.096)	0.67 [0.55 - 0.81]	p < 0.001
	UVU	Proportion of unvoiced speech over total	0.515 (0.101)	1.67 [1.37 - 2.04]	p < 0.001
	F0msestd	Variation in pitch contour irregularity across voiced segments	-0.355 (0.096)	0.7 [0.58 - 0.85]	p < 0.001
Phonation	avg Shimmer	Average short-term amplitude variation	0.424 (0.093)	1.53 [1.27 - 1.83]	p < 0.001
Articulation	avg DDMFCCon_7	Capture the acceleration of spectral changes in MFCC onset transitions; reflect dynamic articulatory patterns	-0.475 (0.106)	0.62 [0.50 - 0.77]	p < 0.001
	std DDMFCCon_8		0.553 (0.1)	1.74 [1.43 - 2.11]	p < 0.001
	avg DF2	Average rate of change in the second formant	-0.475 (0.101)	0.62 [0.51 - 0.76]	p < 0.001
	avg BBEon_2	Average energy in lower Bark bands during speech onsets; reflect vocal fold tension and vowel quality	-0.395 (0.096)	0.67 [0.56 - 0.81]	p < 0.001
	avg BBEon_3		-0.365 (0.097)	0.69 [0.57 - 0.84]	p < 0.001
	avg BBEon_4		-0.294 (0.094)	0.75 [0.62 - 0.90]	0.002
	avg MFCCoff_4	Average MFCC value in offset transitions related to lower formants; reflects vowel quality and articulatory dynamics at speech endings	-0.374 (0.096)	0.69 [0.57 - 0.83]	p < 0.001
	avg MFCCoff_6	Average MFCC value in higher frequencies during speech offsets; captures fine spectral details	-0.349 (0.095)	0.71 [0.59 - 0.85]	p < 0.001
	avg MFCCon_2	Capture lower formant information. influencing vowel quality and speaker identity.	-0.313 (0.097)	0.73 [0.60 - 0.89]	0.002
	avg MFCCon_6	Reflect higher frequency details. important for consonant articulation and fine phonetic distinctions.	-0.305 (0.094)	0.74 [0.61 - 0.89]	0.002
	std DMFCCon_8	Reflect changes in higher frequency details	0.426 (0.097)	1.53 [1.27 - 1.85]	p < 0.001
Phonological	close_mean	Average acoustic features of close vowels (/i/. /u/)	-0.292 (0.103)	0.75 [0.61 - 0.91]	0.005
	stop_mean	Average acoustic features of stop consonants (/p/, /b/, /t/, /k/, /g/, /tʃ/, /d/)	0.43 (0.096)	1.54 [1.28 - 1.85]	p < 0.001
	nasal_mean	Average acoustic features of nasal sounds (/m/, /n/)	-0.427 (0.097)	0.65 [0.54 - 0.79]	p < 0.001
	anterior_mean	Average acoustic features of front vowels	-0.253 (0.093)	0.78 [0.65 - 0.93]	0.006
	close_std	Variability in acoustic features of close vowels (/i/, /u/)	-0.407 (0.106)	0.67 [0.54 - 0.82]	p < 0.001

### Males

For male participants, stress levels were significantly associated with 25 acoustic features spanning all domains (Table 3). In the prosody domain, higher stress was linked to a lower average pitch (F0avg, OR = 0.72) and reduced proportions of voiced speech (VVU, OR = 0.79), alongside increased ratios of unvoiced speech and silence (UP, OR = 1.71). These changes indicate shifts in vocal control and increased vocal tension in response to stress. In the phonation domain, stress was associated with increased shimmer variability (std Shimmer, OR = 1.25), suggesting less stable voice production. Average energy during voiced segments also increased (avgEvoiced, OR = 1.30), possibly reflecting compensatory effort. The articulation domain showed consistent reductions in spectral and temporal variability. Features such as avg DF2 (OR = 0.68), avg MFCCon_2 (OR = 0.68), and std BBEoff_10 (OR = 0.64) indicated reduced articulatory precision and dynamics in both onset and offset of speech. However, features like std DDMFCCoff_4 and std DDMFCCon_5 (both OR = 1.40) suggested increased variability in the acceleration of formant changes, potentially reflecting instability during speech transitions. In the phonological domain, stress led to shorter and less variable pauses (pause_mean, OR = 0.52; pause_std, OR = 0.76). Acoustic variation in lateral sounds decreased (lateral_std, OR = 0.71), and variability in close vowels also declined (close_std, OR = 0.67), pointing to reduced clarity in articulatory targets. Meanwhile, feature related to trills (trill_mean, OR = 1.40) increased, potentially due to greater articulatory tension or effort.

**Table 3:** Voice features associated with stress in men with diabetes.

Category	Feature name	Feature meaning	Coefficient (SD)	Odds Ratio [CI 95%]	PValue (FDR corrected)
Prosody	F0avg	Average fundamental frequency; reflects overall pitch level	-0,33 (0.112)	0.72 [0.58 - 0.90]	0.006
	UP	Ratio of unvoiced segments to pauses	0.535 (0.116)	1.71 [1.36 - 2.14]	p < 0.001
	VVU	Proportion of voiced segments over total speech	-0.236 (0.107)	0.79 [0.64 - 0.97]	0.30
	stdlastEvoiced	Variation in energy at the end of voiced segments	-0.529 (0.125)	0.59 [0.46 - 0.75]	p < 0.001
	avgEvoiced	Average energy in voiced segments	0.259 (0.105)	1.3 [1.05 - 1.59]	0.017
	stddurvoiced	Variation in voiced segment durations	-0.305 (0.113)	0.74 [0.59 - 0.92]	0.010
	PVU	Proportion of pauses relative to total speech	-0.477 (0.118)	0.62 [0.49 - 0.78]	p < 0.001
	avgdurpause	Average duration of pauses	-0.52 (0.116)	0.59 [0.47 - 0.75]	p < 0.001
	PU	Ratio of pauses to unvoiced segments	-0.725 (0.128)	0.48 [0.38 - 0.62]	p < 0.001
Phonation	std Shimmer	Variation in shimmer across voiced segments	0.22 (0.109)	1.25 [1.01 - 1.54]	0.044
Articulation	avg DF2	Average rate of change in the second formant	-0.39 (0.113)	0.68 [0.54 - 0.84]	0.001
	avg BBEoff_6	Average energy in a specific frequency band at the end of speech	0.352 (0.117)	1.42 [1.13 - 1.79]	0.003
	std BBEoff_10	Variation in energy around formants F2-F3 at the end of speech	-0.453 (0.118)	0.64 [0.50 - 0.80]	p < 0.001
	avg MFCCon_2	Average MFCCs during speech onset reflecting lower formants	-0.387 (0.111)	0.68 [0.55 - 0.84]	0.001
	avg MFCCon_4		-0.354 (0.106)	0.7 [0.57 - 0.86]	0.001
	avg MFCCoff_6	Average MFCC value in higher frequencies at speech endings	-0.523 (0.109)	0.59 [0.48 - 0.73]	p < 0.001
	std DMFCCoff_4	Measure changes in lower formants at speech endings	0.322 (0.112)	1.38 [1.11 - 1.72]	0.004
	avg DMFCCoff_5		-0.527 (0.113)	0.59 [0.47 - 0.74]	p < 0.001
	std DDMFCCoff_4	Variation in the acceleration of lower formant changes at speech endings	0.334 (0.113)	1.4 [1.12 - 1.74]	0.003
	std DDMFCCon_5	Variation in the acceleration of lower formant changes at speech onset	0.336 (0.107)	1.4 [1.14 - 1.73]	0.002
Phonological	pause_std	Variation in pause duration	-0.277 (0.107)	0.76 [0.61 - 0.94]	0.012
	pause_mean	Average duration of silence periods	-0.646 (0.115)	0.52 [0.42 - 0.66]	p < 0.001
	lateral_std	Variation in acoustic features of lateral sounds	-0.338 (0.119)	0.71 [0.56 - 0.90]	0.007
	trill_mean	Average acoustic features of trill sounds	0.339 (0.119)	1.4 [1.11 - 1.77]	0.007
	close_std	Variation in acoustic features of close vowels	-0.397 (0.131)	0.67 [0.52 - 0.87]	0.007

### Voice features associated with diabetes distress

#### Females.

For female participants, diabetes distress was significantly associated with one feature in the articulatory domain (Table 4). Specifically, greater distress was linked to reduced variation in the acceleration of lower formant changes at the end of speech (std DDMFCCoff_5, OR = 0.63). This reduction suggests less dynamic modulation in articulatory movements during speech offset, potentially reflecting decreased flexibility under this emotional strain.

**Table 4:** Voice features associated with diabetes distress in women.

Category	Feature name	Feature meaning	Coefficient (SD)	Odds Ratio [CI 95%]	PValue (FDR corrected)
Articulation	std DDMFCCoff_5	Variation in the acceleration of lower formant changes at speech endings	-0.469 (0.101)	0.63 [0.51 - 0.76]	p < 0.001

#### Males.

For male participants, diabetes distress was significantly associated with 18 voice features across prosody, phonation, and articulation domains (Table 5). In the prosodic domain, higher distress levels were linked to increased average pitch tilt across voiced segments (F0tiltavg, OR = 1.75), indicating a more exaggerated intonation pattern. At the same time, reduced variability in pitch movement (F0tiltstd, OR = 0.71) and decreased pitch variation at speech onset (1F0std, OR = 0.68) suggest less dynamic prosodic modulation, possibly reflecting a more rigid speaking under distress. Within the phonation domain, distress was associated with a higher average rate of pitch change (avg DF0, OR = 1.29) and increased shimmer (avg Shimmer, OR = 1.43), both of which may indicate elevated vocal instability. The articulation domain showed the most extensive pattern of associations. Several features related to formant acceleration and high-frequency spectral details, such as avg DDF1 (OR = 1.46), std MFCCon_8 (OR = 1.40), and std DMFCCoff_6 (OR = 1.52), increased with distress, suggesting more intense articulatory movements. However, a few features demonstrated negative associations, including reduced energy in higher-frequency components at speech offset (avg DMFCCoff_8, OR = 0.53) and lower variability in articulatory transitions (std DDMFCCoff_7, OR = 0.67; std DDMFCCon_2, OR = 0.74), which may reflect reductions in articulatory flexibility. Overall, these findings point to a complex articulatory response to distress in men, involving both heightened movement in some aspects and diminished variability in others.

**Table 5:** Voice features associated with diabetes distress in men.

Category	Feature name	Feature meaning	Coefficient (SD)	Odds Ratio [CI 95%]	PValue (FDR corrected)
Prosody	F0tiltavg	Average pitch tilt across voiced segments	0.56 (0.132)	1.75 [1.35 - 2.27]	p < 0.001
	F0tiltstd	Variation in pitch movement trends across voiced segments	-0.338 (0.124)	0.71 [0.56 - 0.91]	0.009
	1F0std	Variation in pitch at the start of speech	-0.383 (0.121)	0.68 [0.54 - 0.86]	0.003
Phonation	avg DF0	Average rate of pitch change over time	0.256 (0.117)	1.29 [1.03 - 1.63]	0.003
	avg Shimmer	Average short-term variation in vocal intensity	0.357 (0.116)	1.43 [1.14 - 1.79]	0.004
Articulation	avg BBEon_1	Average energy in Bark band 1 at speech onset	0.391 (0.118)	1.48 [1.17 - 1.86]	0.003
	avg BBEon_18	Average high-frequency energy at speech onset	0.267 (0.116)	1.31 [1.04 - 1.64]	0.03
	avg DDF1	Average acceleration of the first formant (F1)	0.379 (0.119)	1.46 [1.16 - 1.85]	0.004
	std MFCCon_8	Capture variation and average spectral details in higher frequencies at speech onset	0.337 (0.115)	1.4 [1.12 - 1.75]	0.007
	avg MFCCon_9		0.406 (0.114)	1.5 [1.20 - 1.88]	0.002
	std DMFCCon_11	Variation in the rate of high-frequency spectral changes at speech onset	0.31 (0.119)	1.36 [1.08 - 1.72]	0.02
	std DMFCCoff_6	Capture changes in high-frequency details during speech endings	0.418 (0.118)	1.52 [1.21 - 1.91]	0.002
	avg DMFCCoff_8		-0.637 (0.12)	0.53 [0.42 - 0.67]	p < 0.001
	std DDMFCCon_2	Variation in the acceleration of lower formant changes at speech onset	-0.302 (0.12)	0.74 [0.58 - 0.94]	0.02
	std DDMFCCoff_6	Capture acceleration in high-frequency spectral changes at speech endings	0.395 (0.119)	1.48 [1.18 - 1.87]	0.003
	std DDMFCCoff_7		-0.395 (0.119)	0.67 [0.53 - 0.85]	0.003
	std DDMFCCoff_11		0.361 (0.121)	1.44 [1.13 - 1.82]	0.007
	avg DDMFCCoff_12		0.295 (0.115)	1.34 [1.07 - 1.68]	0.03

### Differences and similarities

When comparing the voice features associated with stress and diabetes distress in female participants, stress was significantly linked to a set of 25 acoustic features spanning all domains (Table 2). These included reductions in pitch and energy variability, changes in voiced segment structure, increased shimmer, and altered articulation of vowels and consonants, indicating that stress broadly affects how speech is produced and modulated. In contrast, diabetes distress was associated with a single articulatory feature (Table 4): reduced variation in the acceleration of lower formant changes at the end of speech. This suggests more localized and subtle articulatory changes under distress. Despite some overlap in the articulation domain affected, no voice feature was significantly associated with both stress and distress. This divergence implies that although both conditions may influence vocal output, they likely do so through distinct physiological or behavioral pathways, leading to different speech alteration patterns. As a sensitivity analysis, highly correlated features (correlation > 0.9) were removed, and results remained consistent, with similar features identified and comparable effect sizes.

For male participants, stress and diabetes distress were each associated with a wide range of acoustic features, spanning all four vocal domains (Tables 3 and 5). However, the specific patterns of association differed between the two. Stress was linked to 25 features, including a lower average pitch, reduced voiced speech, increased shimmer variability, and diminished articulatory precision, indicating a general pattern of reduced vocal control and increased vocal tension. In contrast, diabetes distress was associated with 18 features, particularly in the articulation domain, where both increases and decreases in spectral and formant dynamics were observed. This pattern suggests a more complex articulatory response, involving both heightened effort and reduced flexibility. Despite the shared domain-level involvement, no individual voice feature was significantly associated with both stress and distress. This supports the idea that, while stress and diabetes distress impact overlapping vocal subsystems in men, they likely do so via distinct physiological or behavioral pathways, resulting in different vocal modulation patterns.

Importantly, no common feature were found between male and female participants, whether for stress or for diabetes distress. The absence of shared features between males and females may reflect several factors. From a physiological perspective, sex-related differences in vocal anatomy (e.g., vocal fold length, pitch range, and resonance characteristics) may influence how emotional states such as stress and distress are expressed in speech. Hormonal and behavioral differences may contribute to distinct vocal modulation patterns. Methodological factors may also play a role. Differences in sample size, distribution of stress and distress levels, or variability in speech production could affect feature selection. These findings suggest that voice-based markers of stress and distress may be sex-specific, and highlight the importance of considering sex as a key factor in the development of vocal biomarkers, rather than assuming a single generalizable model.

## Discussion

This work shows that voice characteristics are associated with both stress and diabetes distress in people with diabetes, even after adjusting for key variables such as age, language, diabetes type, and HbA1c. While both emotional states affected vocal features, stress had a broader impact, spanning prosodic, phonatory, articulatory, and phonological domains, whereas diabetes distress showed more limited associations. These findings reflect statistical associations and provide a foundation for future work evaluating predictive or diagnosis applications. The use of aggregated features provides a global representation of vocal behavior, while future work could further investigate approaches that capture temporal dynamics within recordings. Moreover, given the high number of statistical tests performed, False Discovery Rate correction was systematically applied to control for multiple comparisons.

In females with diabetes, stress was significantly associated with 25 voice features spanning prosodic, phonatory, articulatory, and phonological domains, reflecting a broad impact on vocal modulation. These included reduced pitch and energy variability, shorter and more uniform voiced segments, and altered articulation of nasal and vowel sounds, indicating a more constrained and effortful speech pattern under stress. In contrast, diabetes distress in females was associated with only one articulatory feature, reduced variation in formant acceleration at speech offset, suggesting a more localized and subtle impact on articulatory dynamics. The identification of a single feature associated with diabetes distress in women may reflect several factors. First, distress levels were overall moderate in this subgroup, which may have limited statistical power to detect broader associations. Second, it is possible that diabetes distress manifests more subtly in vocal patterns in women, or that the selected features are less sensitive to these changes. These results suggest that voice-based detection of diabetes distress may be more challenging in women and warrant further investigation in larger and more diverse samples. Although both conditions influenced speech production through the articulatory domain, no overlapping features were identified, reinforcing the idea that stress and diabetes distress affect vocal behavior through distinct mechanisms in women. In males with diabetes, both stress and diabetes distress were associated with several acoustic features across all vocal domains, indicating broad and overlapping impacts on vocal mechanisms. Stress was primarily linked to reduced pitch, decreased voiced speech, increased shimmer, and reduced articulatory precision, suggesting a more monotonous and effort-conserving speech style. Diabetes distress was associated with increased pitch tilt and shimmer, along with widespread changes in articulation including both heightened spectral activity and reduced variability in certain transitions, suggesting a more exaggerated and possibly dysregulated vocal effort. Despite the overlap in affected domains, no individual voice feature was shared between stress and distress, pointing again to separate underlying pathways of vocal modulation for each condition in men.

Previous research has shown that stress can influence multiple domains of vocal production, including prosody, phonation, and spectral characteristics. In particular, stress has been associated with increases in fundamental frequency (F0) and pitch variability, reflecting heightened physiological arousal. Changes in phonatory stability, such as increased jitter and shimmer, have also been reported, potentially linked to altered vocal fold tension and control. Additionally, stress may affect speech timing and articulation, leading to modifications in speech rate, pauses, and spectral features. These domain-specific alterations provide a useful framework for interpreting the vocal patterns observed in the present study [42–45]. Our study is consistent with prior literature and extends it by evaluating these associations in a population of people living with diabetes, for whom stress and distress have direct clinical implications for disease management and outcomes. We do not hypothesize that diabetes fundamentally alters the relationship between stress and voice. Rather, this work examines these associations in a population where stress and diabetes-related distress play a central role in health outcomes. Establishing the presence and characteristics of these vocal associations in this specific context is a necessary step toward developing clinically meaningful digital health tools. We show that stress and diabetes distress both influence speech but follow distinct acoustic pathways, and that these vocal features are robust across age, HbA1c levels, language, and type of diabetes. These findings suggest that voice-based features may represent candidate markers of stress and diabetes distress. While further validation is required, voice analysis could potentially contribute to future monitoring approaches when used alongside established assessment tools, with potential for future applications in longitudinal monitoring. Voice-based approaches could be integrated into digital health platforms to support more frequent and low-burden assessment of emotional states. However, their ability to track changes over time and support monitoring applications remains to be established through longitudinal studies. Such approaches may help identify periods of stress or distress and complement existing clinical evaluations. By flagging these states early, healthcare professionals could intervene sooner, and patients could also benefit from self-monitoring and timely support. Such tools may ultimately facilitate more personalized diabetes management and improve overall well-being and medical outcomes. Validated self-report questionnaires such as the PAID remain the reference standard for assessing diabetes-related distress. In this context, voice analysis may offer a complementary approach to capture emotional states in real time.

This study has several strengths. First, it included a large and diverse sample of adults with type 1 and type 2 diabetes, speaking French and English, and with a balanced sex representation. Second, a comprehensive set of acoustic features across multiple speech domains was extracted, and cross-validated statistical methods were used to identify stable associations while controlling for key confounders, including age, HbA1c, diabetes type, and language. Third, collecting voice samples in real-life settings increased the ecological validity of the results, making them more reflective of the everyday vocal behavior of people with diabetes.

However, several limitations should be considered. Despite preprocessing, variability in recording conditions (e.g., background noise, device quality) may have introduced noise, and the use of quality control criteria, while necessary to ensure reliable feature extraction, may limit generalizability to more variable real-world conditions. In addition, participation in a voice-based digital study may introduce selection bias, as individuals who are more comfortable with technology or more motivated to engage in such research may be overrepresented. Technology-related bias may also arise from differences in access to suitable recording devices, and health literacy may influence both participation and the accuracy of self-reported measures. Cultural and linguistic factors, including accent and communication styles, may further influence vocal characteristics and were not explicitly controlled for. Stress and diabetes distress were self-reported and are therefore subject to recall bias and individual interpretation. Moreover, information on comorbid mental health conditions such as depression or anxiety were not included in this work. Both are know to influence self-reported stress and diabetes distress, as well as vocal characteristics, and may have contributed to the observed associations. Future studies should account for these factors to better disentangle their potential effects. In this first study, we deliberately focused on stress and diabetes distress, as both are highly prevalent in people with diabetes, have a direct impact on glycemic control and self-management, and fluctuate dynamically in daily life [15,24,46]. While validated instruments such as the PAID remain the reference standard, their use in this study represents an initial step toward evaluating complementary approaches such as voice-based assessment. Moreover, the use of aggregated features (mean and standard deviation) provides a global representation of vocal behavior but does not capture temporal dynamics within recordings. In addition, although analyses were adjusted for language, we did not perform language-stratified analyses due to sample size constraints. Future studies should investigate language-specific effects and explore feature representations that better preserve temporal structure. Finally, stress and distress levels were overall relatively low in this sample, which may have limited the ability to detect stronger or more extensive associations. Future work should evaluate these findings in more diverse populations and under less controlled conditions, and further investigate the influence of contextual and individual factors on vocal markers.

## Conclusion

This study shows that voice features are associated with stress and diabetes distress in adults living with diabetes and may represent candidate markers for these emotional states. While these emotional states affected similar speech domains, the specific features involved were distinct, suggesting unique vocal signatures for each condition. These associations were robust and independent of age, HbA1c, diabetes type, and language, highlighting their potential generalizability. Given the impact of stress and distress on diabetes management and health outcomes, vocal biomarkers offer a promising complementary approach to traditional self-reporting tools. Beyond monitoring, they could directly inform tailored interventions in clinical and daily life: for instance, flagging patients at risk of elevated distress during routine telehealth visits, triggering personalized digital support, or prompting healthcare providers to initiate timely psychological or behavioral interventions. Integrating voice analysis into digital health platforms could thus not only identify individuals at risk but also support more responsive, personalized care, ultimately improving emotional well-being and reducing diabetes-related complications.

## Abbreviations

PAID: Problem Areas in Diabetes
PSS: Perceived Stress Scale
T1D: Type 1 diabetes
T2D: Type 2 Diabetes

## References

[1] Chu B, Marwaha K, Sanvictores T, Awosika AO, Ayers D. Physiology, stress reaction. StatPearls. 2024. https://www.statpearls.com 31082164

[2] Tsigos C, Kyrou I, Kassi E, Chrousos GP. Stress: Endocrine Physiology and Pathophysiology. In: Feingold KR, Adler RA, Ahmed SF, Anawalt B, Blackman MR, Chrousos G, *et al*., editors. Endotext. South Dartmouth (MA): MDText.com, Inc.; 2020.

[3] Yaribeygi H, Panahi Y, Sahraei H, Johnston TP, Sahebkar A. The impact of stress on body function: a review. EXCLI J. 2017;16:1057–72. doi: 10.17179/excli2017-480 28900385 PMC5579396

[4] Thau L, Gandhi J, Sharma S. Physiology, cortisol. StatPearls. 2023. https://www.statpearls.com 30855827

[5] Satyjeet F, Naz S, Kumar V, Aung NH, Bansari K, Irfan S, et al. Psychological stress as a risk factor for cardiovascular disease: a case-control study. Cureus. 2020;12(10):e10757. doi: 10.7759/cureus.10757 33150108 PMC7603890

[6] Vancheri F, Longo G, Vancheri E, Henein MY. Mental stress and cardiovascular health-part I. J Clin Med. 2022;11(12):3353. doi: 10.3390/jcm11123353 35743423 PMC9225328

[7] Hammen C. Stress and depression. Annu Rev Clin Psychol. 2005;1:293–319. doi: 10.1146/annurev.clinpsy.1.102803.143938 17716090

[8] Morey JN, Boggero IA, Scott AB, Segerstrom SC. Current directions in stress and human immune function. Curr Opin Psychol. 2015;5:13–7. doi: 10.1016/j.copsyc.2015.03.007 26086030 PMC4465119

[9] Alotiby A. Immunology of stress: a review article. J Clin Med. 2024;13(21):6394. doi: 10.3390/jcm13216394 39518533 PMC11546738

[10] Sharma K, Akre S, Chakole S, Wanjari MB. Stress-induced diabetes: a review. Cureus. 2022;14(9):e29142. doi: 10.7759/cureus.29142 36258973 PMC9561544

[11] Merabet N, Lucassen PJ, Crielaard L, Stronks K, Quax R, Sloot PMA, et al. How exposure to chronic stress contributes to the development of type 2 diabetes: a complexity science approach. Front Neuroendocrinol. 2022;65:100972. doi: 10.1016/j.yfrne.2021.100972 34929260

[12] Ingrosso DMF, Primavera M, Samvelyan S, Tagi VM, Chiarelli F. Stress and diabetes mellitus: pathogenetic mechanisms and clinical outcome. Horm Res Paediatr. 2023;96(1):34–43. doi: 10.1159/000522431 35124671

[13] Harris ML, Oldmeadow C, Hure A, Luu J, Loxton D, Attia J. Stress increases the risk of type 2 diabetes onset in women: a 12-year longitudinal study using causal modelling. PLoS One. 2017;12(2):e0172126. doi: 10.1371/journal.pone.0172126 28222165 PMC5319684

[14] Hackett RA, Steptoe A. Type 2 diabetes mellitus and psychological stress - a modifiable risk factor. Nat Rev Endocrinol. 2017;13(9):547–60. doi: 10.1038/nrendo.2017.64 28664919

[15] Franc S, Bensaid S, Schaepelynck P, Orlando L, Lopes P, Charpentier G. Impact of chronic emotions and psychosocial stress on glycemic control in patients with type 1 diabetes. Heterogeneity of glycemic responses, biological mechanisms, and personalized medical treatment. Diabetes Metab. 2023;49(6):101486. doi: 10.1016/j.diabet.2023.101486 37858921

[16] Hilliard ME, Yi-Frazier JP, Hessler D, Butler AM, Anderson BJ, Jaser S. Stress and A1c among people with diabetes across the lifespan. Curr Diab Rep. 2016;16(8):67. doi: 10.1007/s11892-016-0761-3 27287017 PMC4936828

[17] Skinner TC, Joensen L, Parkin T. Twenty-five years of diabetes distress research. Diabet Med. 2020;37(3):393–400. doi: 10.1111/dme.14157 31638279

[18] Poole L, Hackett RA. Diabetes distress: the psychological burden of living with diabetes. Lancet Diabetes Endocrinol. 2024;12(7):439–41. doi: 10.1016/S2213-8587(24)00126-8 38824928

[19] Fisher L, Hessler D, Polonsky W, Strycker L, Masharani U, Peters A. Diabetes distress in adults with type 1 diabetes: prevalence, incidence and change over time. J Diabetes Complications. 2016;30(6):1123–8. doi: 10.1016/j.jdiacomp.2016.03.032 27118163 PMC4949147

[20] Canha D, Aguayo G, Cosson E, Vaduva P, Renard E, Alzaid F, et al. Clinical phenotyping of people living with type 1 diabetes according to their levels of diabetes-related distress: results from the SFDT1 cohort. BMJ Open Diabetes Res Care. 2025;13(1):e004524. doi: 10.1136/bmjdrc-2024-004524 40000027 PMC11865782

[21] Perrin NE, Davies MJ, Robertson N, Snoek FJ, Khunti K. The prevalence of diabetes-specific emotional distress in people with Type 2 diabetes: a systematic review and meta-analysis. Diabet Med. 2017;34(11):1508–20. doi: 10.1111/dme.13448 28799294

[22] Sendekie AK, Limenh LW, Bizuneh GK, Kasahun AE, Wondm SA, Tamene FB, et al. Psychological distress and its impact on glycemic control in patients with diabetes, Northwest Ethiopia. Front Med (Lausanne). 2025;12:1488023. doi: 10.3389/fmed.2025.1488023 40206466 PMC11979121

[23] Hagger V, Hendrieckx C, Cameron F, Pouwer F, Skinner TC, Speight J. Diabetes distress is more strongly associated with HbA1c than depressive symptoms in adolescents with type 1 diabetes: results from Diabetes MILES Youth-Australia. Pediatr Diabetes. 2018;19(4):840–7. doi: 10.1111/pedi.12641 29383803

[24] Park H-S, Cho Y, Seo DH, Ahn SH, Hong S, Suh YJ, et al. Impact of diabetes distress on glycemic control and diabetic complications in type 2 diabetes mellitus. Sci Rep. 2024;14(1):5568. doi: 10.1038/s41598-024-55901-0 38448443 PMC10917807

[25] Wojujutari Ajele K, Sunday Idemudia E. The role of depression and diabetes distress in glycemic control: a meta-analysis. Diabetes Res Clin Pract. 2025;221:112014. doi: 10.1016/j.diabres.2025.112014 39892818

[26] Roots P. Perceived stress scale. Psychology Roots. 2020.

[27] Polonsky WH, Anderson BJ, Lohrer PA, Welch G, Jacobson AM, Aponte JE, et al. Assessment of diabetes-related distress. Diabetes Care. 1995;18(6):754–60. doi: 10.2337/diacare.18.6.754 7555499

[28] Giddens CL, Barron KW, Byrd-Craven J, Clark KF, Winter AS. Vocal indices of stress: a review. J Voice. 2013;27(3):390.e21-9. doi: 10.1016/j.jvoice.2012.12.010 23462686

[29] Van Puyvelde M, Neyt X, McGlone F, Pattyn N. Voice stress analysis: a new framework for voice and effort in human performance. Front Psychol. 2018;9:1994. doi: 10.3389/fpsyg.2018.01994 30515113 PMC6255927

[30] Besser A, Lotem S, Zeigler-Hill V. Psychological stress and vocal symptoms among university professors in Israel: implications of the shift to online synchronous teaching during the COVID-19 pandemic. J Voice. 2022;36(2):291.e9-291.e16. doi: 10.1016/j.jvoice.2020.05.028 32600872 PMC7274605

[31] Lugger M, Yang B. The relevance of voice quality features in speaker independent emotion recognition. In: 2007 IEEE International Conference on Acoustics, Speech and Signal Processing - ICASSP ’07, 2007. IV-17-IV–20. 10.1109/icassp.2007.367152

[32] Kaur S, Kulkarni N. A deep learning technique for emotion recognition using face and voice features. In: 2021 IEEE Pune Section International Conference (PuneCon), 2021. 1–6. 10.1109/punecon52575.2021.9686510

[33] Home. Colive Voice. 2022. Accessed 2025 May 6. https://www.colivevoice.org/en/

[34] Dehak N, Dumouchel P, Kenny P. Modeling prosodic features with joint factor analysis for speaker verification. IEEE Trans Audio Speech Lang Process. 2007;15(7):2095–103. doi: 10.1109/tasl.2007.902758

[35] Vásquez-Correa JC, Orozco-Arroyave JR, Bocklet T, Nöth E. Towards an automatic evaluation of the dysarthria level of patients with Parkinson’s disease. J Commun Disord. 2018;76:21–36. doi: 10.1016/j.jcomdis.2018.08.002 30149241

[36] Arias-Vergara T, Vásquez-Correa JC, Orozco-Arroyave JR. Parkinson’s disease and aging: analysis of their effect in phonation and articulation of speech. Cognit Comput. 2017;9:731–48.

[37] Vásquez-Correa JC, Klumpp P, Orozco-Arroyave JR, Nöth E. Phonet: a tool based on gated recurrent neural networks to extract phonological posteriors from speech. In: Interspeech 2019, 2019. 549–53. 10.21437/interspeech.2019-1405

[38] Orozco-Arroyave JR, Vásquez-Correa JC, Vargas-Bonilla JF, Arora R, Dehak N, Nidadavolu PS, et al. NeuroSpeech: an open-source software for Parkinson’s speech analysis. Digital Signal Process. 2018;77:207–21. doi: 10.1016/j.dsp.2017.07.004

[39] Lausen A, Schacht A. Gender differences in the recognition of vocal emotions. Front Psychol. 2018;9:882. doi: 10.3389/fpsyg.2018.00882 29922202 PMC5996252

[40] Schirmer A, Striano T, Friederici AD. Sex differences in the preattentive processing of vocal emotional expressions. Neuroreport. 2005;16(6):635–9. doi: 10.1097/00001756-200504250-00024 15812323

[41] Meinshausen N, Bühlmann P. Stability selection. J R Stat Soc Series B Stat Methodol. 2010;72:417–73.

[42] Larrouy-Maestri P, Poeppel D, Pell MD. The sound of emotional prosody: nearly 3 decades of research and future directions. Perspect Psychol Sci. 2024. doi: 17456916231217722 10.1177/17456916231217722 PMC12231869 38232303

[43] Cohen AS, Hong SL, Guevara A. Understanding emotional expression using prosodic analysis of natural speech: refining the methodology. J Behav Ther Exp Psychiatry. 2010;41(2):150–7. doi: 10.1016/j.jbtep.2009.11.008 20022000

[44] Laukka P, Linnman C, Åhs F, Pissiota A, Frans Ö, Faria V, et al. In a nervous voice: acoustic analysis and perception of anxiety in social phobics’ speech. J Nonverbal Behav. 2008;32(4):195–214. doi: 10.1007/s10919-008-0055-9

[45] Tolkmitt FJ, Scherer KR. Effect of experimentally induced stress on vocal parameters. J Exp Psychol Hum Percept Perform. 1986;12(3):302–13. doi: 10.1037/0096-1523.12.3.302 2943858

[46] Misra R, Shawley-Brzoska S, Khan R, Kirk BO, Wen S, Sambamoorthi U. Addressing diabetes distress in self-management programs: results of a randomized feasibility study. J Appalach Health. 2021;3(3):68–85. doi: 10.13023/jah.0303.06 35770030 PMC9192112

